# Theory of Magnetic Properties in Quantum Electrodynamics Environments: Application to Molecular Aromaticity

**DOI:** 10.1021/acs.jctc.4c00195

**Published:** 2024-09-10

**Authors:** Alberto Barlini, Andrea Bianchi, Enrico Ronca, Henrik Koch

**Affiliations:** † Scuola Normale Superiore, Pisa 56126, Italy; ‡ Dipartimento di Chimica, Biologia e Biotecnologie, Università degli Studi di Perugia, Perugia 06123, Italy; § Department of Chemistry, Norwegian University of Science and Technology, Trondheim 7491, Norway

## Abstract

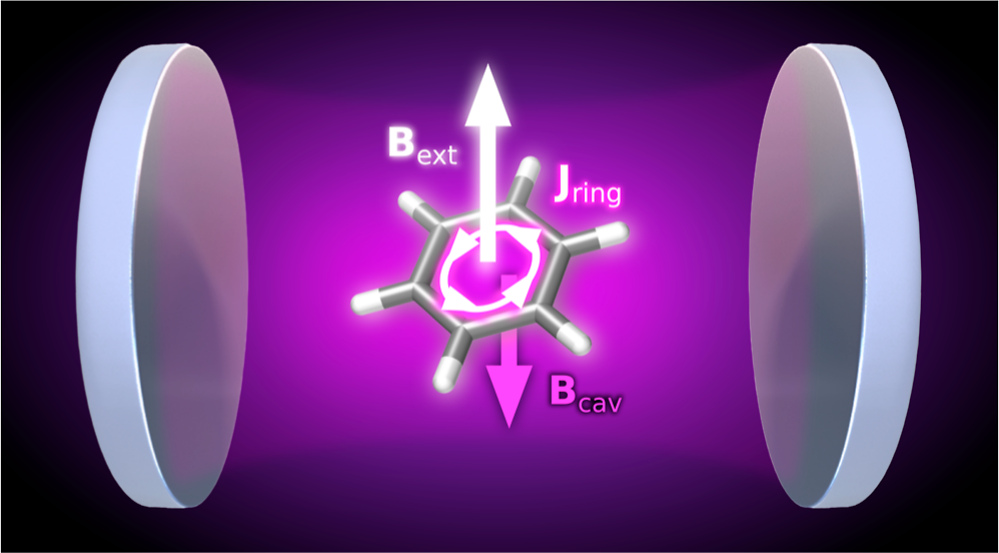

In this work, we present ab initio cavity quantum electrodynamics (QED) methods which include interactions with a static magnetic field and nuclear spin degrees of freedom using different treatments of the quantum electromagnetic field. We derive explicit expressions for QED-HF magnetizability, nuclear shielding, and spin–spin coupling tensors. We apply this theory to explore the influence of the cavity field on the magnetizability of saturated, unsaturated, and aromatic hydrocarbons, showing the effects of different polarization orientations and coupling strengths. We also examine how the cavity affects aromaticity descriptors, such as the nucleus-independent chemical shift and magnetizability exaltation. We employ these descriptors to study the trimerization reaction of acetylene to benzene. We show how the optical cavity induces modifications in the aromatic character of the transition state leading to variations in the activation energy of the reaction. Our findings shed light on the effects induced by the cavity on magnetic properties, especially in the context of aromatic molecules, providing valuable insights into understanding the interplay between the quantum electromagnetic field and molecules.

## 1 Introduction

Polaritonic chemistry has recently gained significant attention, thanks to pioneering research by Ebbesen et al.,^1^ which demonstrated that the strong-light matter coupling can influence photochemical reactions and ground state reactivity.^2−4^ Several experimental findings have revealed the influence of electromagnetic confinement on a wide range of processes, including chemical reactions,^1−10^ singlet fission,^11−13^ intersystem crossing,^14−16^ and crystallization,^17−19^ as well as optical properties such as absorption, scattering, and emission.^20−36^

Recently, experimental works have reported the effect of a quantum electromagnetic field on molecular magnetic properties. Eddins et al.^37^ reported the strong coupling of molecular nanomagnets within a microwave cavity. Ghirri et al.^38^ developed devices that operate in the microwave range in the presence of strong magnetic fields. These devices have been used to couple photon and electronic spin degrees of freedom, showing potential applications in quantum information.^39^ Jenkins et al.^40^ proposed a magnetic quantum processor composed of individual molecular spins coupled to superconducting coplanar resonators. Not only the field effects on the magnetic properties of matter have been investigated. Recently Ebbesen et al.^41^ explored standard nuclear magnetic resonance (NMR) spectroscopy as a tool to investigate vibrational strong coupling effects inside microfluidic optical cavities.

From a theoretical perspective, extensive progress has been made in recent years to describe the physical states of strongly light-matter coupled systems. Various ab initio quantum electrodynamics (QED) approaches have emerged, including QED density functional theory (QEDFT),^42,43^ QED Hartree–Fock (QED-HF),^44,45^ strong coupling QED Hartree–Fock (SC-QED-HF),^46^ second-order QED Møller–Plesset perturbation theory (QED-MP2),^47^ QED coupled cluster (QED-CC),^44^ QED full configuration interaction (QED-FCI),^44^ and more.^48−51^ Recently, Rokaj et al.^52^ proposed a theory for describing the interaction of solid-state materials coupled to a quantum electromagnetic field and a static external magnetic field of arbitrary strength. However, there are currently no theoretical studies on the quantum field effects on the magnetic properties of molecules. These properties involve magnetizability, defined as the second derivative of the energy with respect to an external magnetic field,^53^ nuclear shielding, and indirect spin–spin couplings, both of which play a key role in simulations of NMR spectroscopy.^54^ Moreover, both magnetizability and nuclear shielding tensors, are employed as aromaticity descriptors for molecules^55−57^ and even aromatic transition states.^58−64^ Specifically, the nucleus-independent chemical shift (NICS) serves as a quantitative and qualitative gauge of the induced magnetic field within a molecule in an external magnetic field.^65^ In addition, the magnetizability exaltation quantifies the increase in magnetizability due to the electron delocalization associated with ring currents.^66^

In this paper, we developed ab initio methods to investigate quantum field-induced effects on magnetic properties. In the first part of the paper, a general theory based on the minimal coupling Hamiltonian is presented. In Section 2.2, the Hamiltonian with an approximate description of the cavity field that extends beyond the dipole approximation is derived. In Section 2.3, the dipole approximation is introduced, and the dipolar Hamiltonian in the length gauge form is derived. In Section 2.4, the expressions of the dipolar Hamiltonian derivatives are given. In Section 2.5, the dipolar Hamiltonian is used to derive QED-HF expressions to simulate magnetizabilities, nuclear shieldings, and indirect spin–spin couplings. In the last part of the paper, we applied our implementation to investigate the effects of the quantum field. In Section 4.1, the results for the magnetizabilities of saturated, unsaturated, and aromatic hydrocarbons are presented. In Section 4.2, the calculations of NICS and magnetizability exaltation are reported. Finally, our concluding remarks are given in Section 5.

## 2 Theory

In the upcoming sections, we will start from a QED minimal coupling Hamiltonian in the presence of a static magnetic field.^52^ Then, we will derive a QED Hamiltonian with an approximated cavity field that goes beyond the dipole approximation. We will formulate the dipolar Hamiltonian and we will report its derivatives. Lastly, we will derive expressions for the QED-HF magnetizability, nuclear shieldings, indirect spin–spin couplings, and their response equations.

### 2.1 QED Hamiltonian with a Static Magnetic Field

In the Born–Oppenheimer approximation, the radiation–matter interaction can be described in the nonrelativistic limit by the minimal coupling Hamiltonian,^67^ which in atomic units reads as
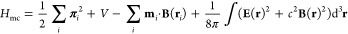
1where **E**(**r**) and **B**(**r**) are the electric and magnetic fields of the cavity, and *V* is the electrostatic potential. The kinetic momentum operator π_*i*_ for the electron *i* is given as
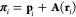
2

In eq 2, **p**_*i*_ is the momentum operator, and **A**(**r**_*i*_) is the vector potential associated with the quantum electromagnetic field
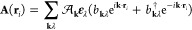
3

The operators *b*_**k**λ_^†^ and *b*_**k**λ_ create and annihilate a photon with frequency ω_**k**_, wave vector **k**, and polarization **ε**_λ_, respectively. The coupling strength is
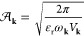
4where the vacuum permittivity is equal to 1/4π in atomic units, ε_r_ is the relative permittivity, and *V*_**k**_ denotes the quantization volume of the mode defined by the wave vector **k**. In eq 1, the electron magnetic moment **m**_*i*_
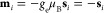
5interacts with the magnetic field of the cavity. Here, *g*_e_ is the electron *g*-factor, μ_B_ is the Bohr magneton, and **s**_*i*_ is the electron spin operator associated with the electron *i*. In the presence of a homogeneous external magnetic field **B**_ext_ described by the external vector potential **A**_ext_, and nuclear magnetic moments **M**_*K*_ that give rise to the vector potential **A**_n_, the Hamiltonian in eq 1 becomes
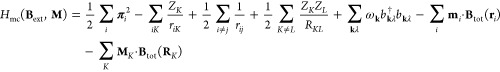
6where **r**_*i*_ and **R**_*K*_ indicate the positions of the electron *i* and the nucleus *K*, respectively. Here, we refer collectively to the magnetic moments by **M** = {**M**_*K*_}. In eq 6, the kinetic momentum operator now includes the following vector potential
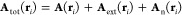
7

The vector potential associated with the static magnetic field is
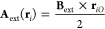
8where *r*_*iO*_ = |**r**_*i*_ – **R**_*O*_| is the distance between the electron *i* and the gauge origin **O**. This term introduces a gauge origin dependence in the Hamiltonian that vanishes in the limit of a complete orbital basis.^54^ With a truncated orbital basis, gauge origin independence is no longer guaranteed. To overcome this problem, we employed London atomic orbitals (LAOs),^68^ as they have been extensively used in gauge origin-independent calculations of molecular magnetic properties.^53,54,69−71^ In eq 7, the vector potential from the nuclear magnetic moments is given by
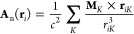
9where *r*_*iK*_ = |**r**_*i*_ – **R**_*K*_| is the distance between the electron *i* and the nucleus *K*. Note that eq 9 is invariant with respect to the choice of the origin. The curl of the vector potential in eq 7 gives the total magnetic field
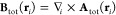
10which is expressed as the sum of the different contributions
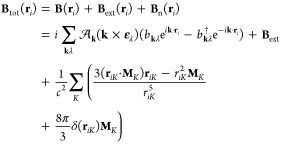
11

The interaction between the nuclear spin degrees of freedom with the total magnetic field is described through the Zeeman interactions in eq 6, where the nuclear magnetic moment **M**_*K*_ is
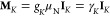
12where *g*_*K*_ is the nuclear *g*-factor, μ_N_ is the nuclear magneton, γ_*K*_ is the magnetogyric ratio, and **I**_*K*_ is the nuclear spin operator associated with the nucleus *K*.

### 2.2 Cavity Field Approximation

As a first approximation, we express the cavity magnetic field **B**(**r**_*i*_) entering the Hamiltonian in eq 6 as
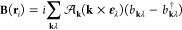
13where we have set exp (±*i***k** · **r**_*i*_) = 1. Note that eq 13 differs from the usual dipole approximation, where the magnetic field of the cavity is zero since the cavity vector potential is set to **A**(**r**) = **A**(0). To include the magnetic contribution of the quantum field, it would be necessary to go beyond the dipole approximation considering the interactions arising from the magnetic dipole and electric quadrupole.^72^ However, using the approximation in eq 13 enables us to maintain the cavity magnetic dipole interaction terms, avoiding this complication. The associated vector potential to eq 13 is
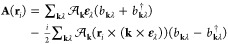
14where the first term is the cavity vector potential in the dipole approximation and the second term gives rise to the cavity magnetic field in eq 13. The corresponding electric field is given by
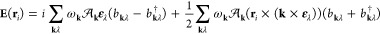
15

It is important to note that eqs 13 and 15 fulfill Maxwell’s equations except for Ampère–Maxwell’s law^73^
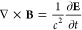
16as in the case of the standard dipole approximation.^72^ In fact, in eq 16, the left-hand side is zero whereas the right-hand side does not vanish because of the time dependence of the photon operators in eq 15. Further details related to this issue are reported in the Supporting Information. Despite this limitation, using the cavity vector potential in eq 14 allows us to include the cavity magnetic dipole interaction terms. The total vector potential now takes the form
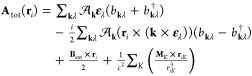
17

The length gauge form of the Hamiltonian is obtained from eq 17 by applying the following transformation
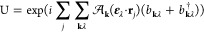
18

The transformed conjugate momentum π_*i*_ = **p**_*i*_ + **A**_tot_(**r**_*i*_) then becomes
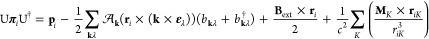
19here, we assumed that the cavity has at least two modes with wave vectors **k** and −**k**, respectively. Therefore, the two-electron terms arising from the transformation of the total vector potential in eq 17 vanish. Using the conjugate momentum in eq 19 and applying the unitary transformation
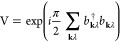
20to change the phases of the photon operators,^72^ we obtain the following Hamiltonian
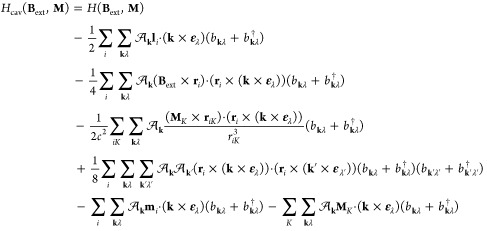
21where **l**_*i*_ = **r**_*iO*_ × **p**_*i*_ is the angular momentum. Here, *H*(**B**_ext_, **M**) represents the dipolar Hamiltonian that will be examined in the following section. The above transformation introduces new interaction terms that couple the external magnetic field, the electron spin, and the nuclear magnetic moments with the magnetic field of the cavity.

### 2.3 Dipolar Hamiltonian

To further simplify the Hamiltonian in eq 21, we apply the dipole approximation by assuming that the relevant electromagnetic modes have a wavelength much larger than the characteristic lengths of the molecules. Disregarding the terms that are linear or higher in |**k**|, we obtain the dipole Hamiltonian in the length gauge representation
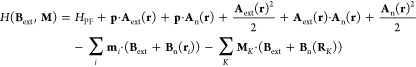
22here, *H*_PF_ is the standard Pauli–Fierz Hamiltonian^72^
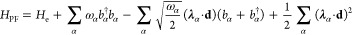
23where *H*_e_ is the standard electronic Hamiltonian, **d** is the total dipole moment, and **λ**_α_ the polarization vector, which are indicated as
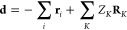
24
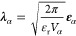
25respectively. In eq 23, we introduced α to denote the photonic mode defined by the wave vector **k** and polarization **ε**_λ_. It is important to note that the Hamiltonian in eq 22 depends also on the choice of the origin of the multipole expansion. However, origin invariance can be explicitly imposed by a suitable unitary transformation, as shown in ref (44). In the second quantization the Hamiltonian in eq 22 may be written as
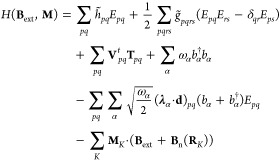
26where *p*, *q*, *r*, and *s* denote the molecular orbitals. In eq 26, we have introduced the singlet excitation operators
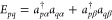
27and the triplet excitation operators, which in the Cartesian representation read as
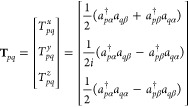
28here, we have disregarded the dependence of the operators on the magnetic field since our focus is on calculating molecular properties as energy derivatives. Thus, orbital connection schemes can be employed to avoid operator dependence.^74^ Further details concerning the derivations of molecular properties expressions using orbital connection schemes will be given in the next section. The one-electron integrals in eq 26 include the one-electron dipole self-energy contribution and the first- and second-order singlet corrections due to the external fields
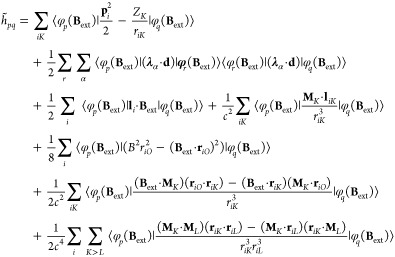
29where **l**_*iK*_ = **r**_*iK*_ × **p**_*i*_ is the angular momentum around the nucleus *K*. The two-electron integrals now also include the two-electron dipole self-energy contribution
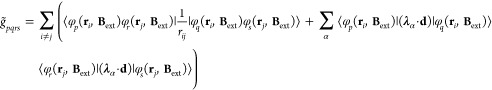
30

The first-order triplet corrections arising from the interaction of the electron spin with the external magnetic field and the nuclear magnetic dipole moments are collected in
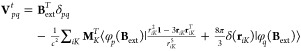
31

A complete description of the interactions represented by the integrals in eqs 29–31 will be given in the next section. Note that in eqs 29–31 the MOs are field-dependent as they are expanded over the LAO basis. The LAOs are defined as
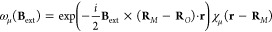
32where χ_μ_ is an atomic orbital centered on nucleus *M* at position **R**_*M*_. This choice of basis ensures a gauge-origin independent description of the atomic system in a finite basis set calculation.

### 2.4 Derivatives of the Dipolar Hamiltonian

The Hamiltonian in eq 26 is valid at all values of **B**_ext_ and **M**. Since the interactions with the external magnetic field and the nuclear magnetic dipole moments are much smaller than those in chemical bonds, we may now expand the Hamiltonian in **B**_ext_ and **M** at **B**_ext_ = **0** and **M** = **0**
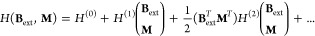
33where the indices in the parentheses (*n*) denote the *n*-th order derivative with respect to **B**_ext_ and **M**. The zeroth-order Hamiltonian corresponds to the Pauli–Fierz Hamiltonian in eq 23. The first-order Hamiltonian describes the paramagnetic interactions
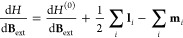
34
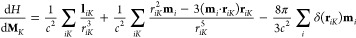
35where the first term arises from the dependence of the atomic orbitals on the static magnetic field. The second term couples the orbital motion and the static magnetic field, and the last term arises from the electronic Zeeman interaction. In eq 35, the first term represents the paramagnetic spin–orbit coupling. The last two terms correspond to spin-dipole and Fermi-contact interactions, which couple the nuclear magnetic moments to the electron spin. The second-order interaction terms read as
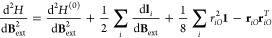
36
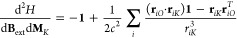
37
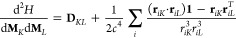
38which correspond to the common diamagnetic interactions.^54,74^ The purely nuclear contribution in eq 37 arises from the nuclear Zeeman interaction, while in eq 38, it originates from the classical dipolar interaction, where **D**_*KL*_ is
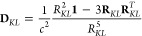
39

To construct the field-dependent molecular orbitals in eq 26, we employed the symmetric orbital connection proposed by Helgaker and Jørgensen.^74^ In this formalism, we require the MOs to stay orthonormal for any value of the perturbing field. The set of orthonormalized molecular orbitals (OMOs) is written as
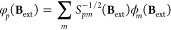
40where
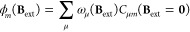
41are the so-called unmodified molecular orbitals (UMOs), obtained by combining London atomic orbitals using the zero-field coefficients. In eq 40, we used the shorthand notation , where *S*^UMO^ is the overlap matrix in the UMOs basis. The OMOs in eq 40 are such that their derivative with respect to the magnetic field is
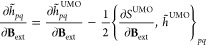
42where the curly brackets represent the one-index transformed integrals
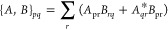
43and similarly in the case of the two-electron integrals.^75^ Therefore, we can express the *n*-th order derivative of the Hamiltonian in eq 26 in terms of the UMOs. However, the contribution from the reorthonormalization of the molecular orbitals must be included, as shown in eq 42. For more details about orbital connections, we refer to these extended discussions in the literature.^74−77^

### 2.5 QED-HF Magnetic Properties

In the QED-HF model, the wave function ansatz is given as
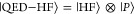
44

Here, |HF⟩ represents a single Slater determinant, and |*P*⟩ is
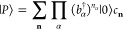
45where |0⟩ denotes the photonic vacuum state, and *c*_**n**_ are the coefficients describing the expansion of photon number states. In the absence of external fields, the energy can be minimized with respect to the photon coefficients for a given HF state. This can be achieved by diagonalizing the photonic Hamiltonian
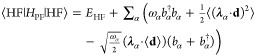
46through a unitary coherent-state transformation^44^
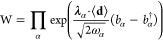
47where ⟨**d**⟩ is given by
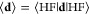
48with **d** being the total dipole moment operator. The orbitals in the HF reference are optimized with an orthogonal transformation, defined as exp(−κ), where κ is an antisymmetric one-electron operator. In the coherent-state basis, the reference wave function is written as
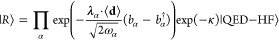
49allowing the energy calculation to remain invariant with respect to the choice of the origin, even for charged molecules. Consequently, the polaritonic properties obtained through analytical energy derivatives are independent of the multipole expansion origin. In the presence of the external fields, the QED-HF energy may be written as
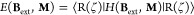
50where ζ represents the optimized values of both electronic and photonic parameters, that satisfy the variational condition
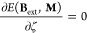
51for all values of **B**_ext_ and **M**. Note that eq 51 determines the implicit dependence of the parameters ζ on the perturbations **B**_ext_ and **M**. In addition, as the QED-HF method is variational, we can employ the standard procedure for variational wave functions to derive the expression of the polaritonic properties as analytical derivatives of the energy. The magnetic properties can be defined via the second-order derivatives as^54^
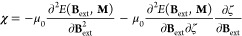
52
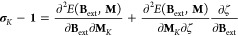
53
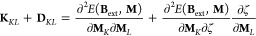
54where **χ** is the magnetizability tensor and μ_0_ is the magnetic permeability of free space, **σ**_*K*_ is the nuclear shielding tensor referred to the nucleus *K*, and **K**_*KL*_ is the indirect nuclear spin–spin coupling tensor between the nuclei *K* and *L*. These second-order derivatives require only the first-order parameters with respect to the magnetic field **B**_ext_ or the nuclear dipole moment **M**_*L*_. The first-order parameters are obtained from the variational condition eq 51 by taking the derivatives with respect to **B**_ext_ and **M**_*K*_, and they read
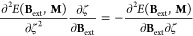
55
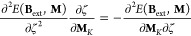
56

These linear systems of equations can be solved iteratively and they require the first-order derivative with respect to the perturbation of the polaritonic energy gradient and the polaritonic energy Hessian. To derive explicit expressions for the QED-HF energy derivatives, we first express the Hamiltonian in eq 26 in the coherent-state basis
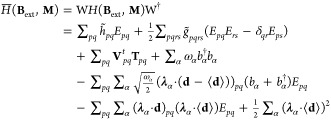
57

For the magnetizability and the nuclear shieldings, the first-order response to the magnetic field is required, which is described by the imaginary part of the first-order parameters. Therefore, the QED-HF wave function can be parametrized as follows
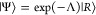
58where the operator Λ may be chosen as
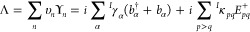
59and the operator *E*_*pq*_^+^ is given by
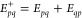
60

The transformation in eq 59 is necessary to introduce photon and electronic parameters to describe the first-order response of the QED-HF wave function. Here, γ_α_ describes the response of the coherent state to the perturbations, whereas κ_*pq*_ represents the response of the orbitals including only nonredundant parameters. Following the general theory presented in ref (72), we may write eqs 52, and 53 as
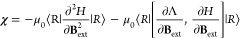
61
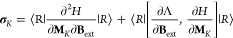
62and the response equations in eq 55 as
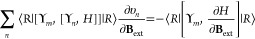
63where the left-hand side includes the polaritonic energy Hessian and the first-order response of the polaritonic wave function, while the right-hand side is given by the first-order derivative of the polaritonic energy gradient with respect to **B**_ext_. Note that the elements of the Hessian on the left-hand side vanish for the coupling between electronic and photonic degrees of freedom. Similarly, the right-hand side is zero for the photonic operators as no terms couple the magnetic field **B**_ext_ with photonic degrees of freedom in the Hamiltonian. This lack can be attributed to the use of the dipole approximation in describing light-matter interaction. The linear system of equations reduces to
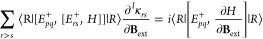
64

This set of equations is similar to the HF magnetic response equations,^53^ except for the presence of dipole self-energy terms on both sides, which will thus affect the response of the orbitals to the magnetic field. Moreover, new contributions from the dipole self-energy also arise in eqs 61 and 62 due to the use of London atomic orbitals. The derivatives of the one-electron integrals are
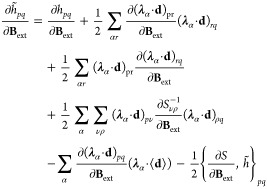
65where we used the notation . Note that in eq 65, the derivatives of the dipole operator and the inverse of the overlap matrix are also required. The derivatives of the two-electron integrals are given by
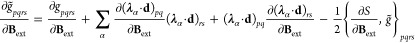
66

Similarly, the second-order derivatives can be obtained following the procedure reported in ref (75). To find explicit expressions for the indirect spin–spin coupling tensor **K**_*KL*_ in eq 54, we need to change the wave function parametrization in eq 59, as the nuclear perturbations involve triplet operators. The operator Λ then may be written as
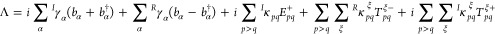
67where the triplet operators are
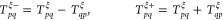
68with ξ labeling a Cartesian component between *x*, *y*, *z*. The expression for the indirect spin–spin coupling is obtained as
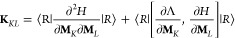
69

The evaluation of this property requires solving the nuclear response equations for the orbital response
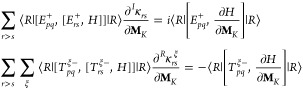
70which now involve the dipole self-energy contributions to the Hessian on the left-hand side. The response equations in eq 70 allow us to find how the orbital parameters are modified by the effect of singlet and triplet nuclear perturbations. The solutions of eq 70 are used in eq 69 to calculate the spin–spin coupling tensors for each couple of nuclei. Despite the inclusion of the quantum field, the explicit expression of the indirect spin–spin coupling remains unchanged. However, the effects of the dipole self-energy are now included in the wave function response.

## 3 Validation and Implementation

The calculation of HF and QED-HF magnetic properties has been implemented in a development version of the eT program.^78^ This implementation follows the standard procedure for calculating molecular magnetic properties.^53,54^ The response equations are solved iteratively using a linear subspace solver to obtain the first-order response of the wave function, which is then employed to calculate the properties. The QED-HF magnetizability and shielding codes have been numerically validated by comparison with QED-HF finite field calculations. The QED-HF spin–spin coupling code has been validated by setting the coupling strength to zero and comparing the results with HF spin–spin couplings. We do not present the results for QED-HF spin–spin coupling as this property is not used in the description of the molecular aromaticity. Furthermore, it is also well-known that the HF approximation is not appropriate for calculating triplet molecular properties, as a correlated method is needed, such as coupled cluster singles and doubles (CCSD).^79^ The development of a CCSD implementation will be reported elsewhere.

## 4 Results and Discussions

All molecular geometries used in this paper have been optimized using the ORCA software package^80^ using a DFT-B3LYP level of theory and a def2-SVP basis set.^81^ These geometries are available in the Supporting Information. All calculations of the magnetic properties reported in this paper have been performed using an aug-cc-pVDZ basis set.^82,83^ In the following sections, we present the QED-HF magnetizabilities for a range of hydrocarbons comparing them with the no-cavity HF values. Additionally, we report the effects of strong light-matter coupling on the aromaticity descriptors as the NICS and magnetizability exaltation used to study the reaction pathway of the acetylene trimerization to benzene in optical cavity.

### 4.1 Modulation of Magnetizabilities

**Figure 1:**
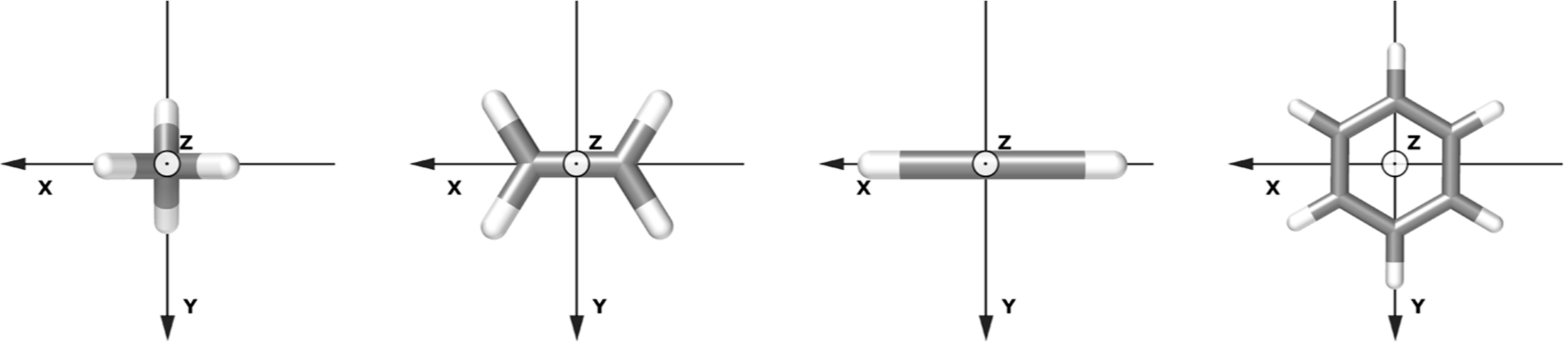
Graphical representation of the investigated molecules, methane, ethylene, acetylene, and benzene.

In this section, we explore the strong light-matter coupling effects on the magnetizabilities of the methane, ethylene, acetylene, and benzene molecules. We examined the effect of different polarization orientations and coupling strength on the isotropic magnetizabilities
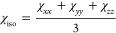
71where χ_*xx*_, χ_*yy*_, and χ_*zz*_ are the diagonal elements of the magnetizability tensor. The QED-HF calculations were conducted within an optical cavity with a coupling strength of 0.1 a.u. The methane molecule was positioned with the carbon atom in the origin and the two couples of protons aligned along the *x*- and *y*-axis, respectively. The ethylene and acetylene were positioned within the cavity with the C–C bonds aligned along the *x*-axis, whereas the benzene molecule was oriented to lie in the *xy*-plane. The three different polarization orientations were chosen along the *x*-, *y*- and *z*-axis, as shown in Figure 1.

**Figure 2:**
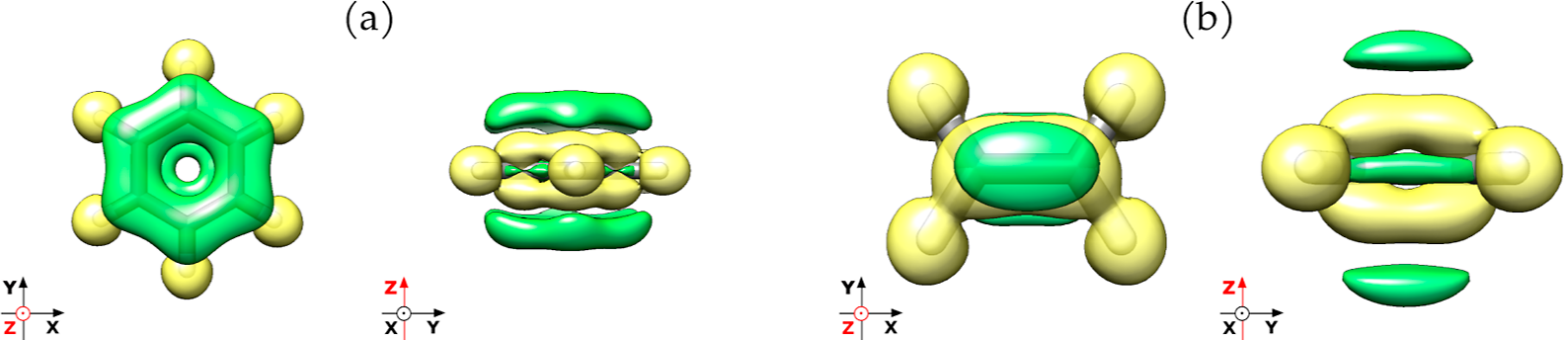
Difference between electron densities computed with λ = 0.05 and λ = 0.00 a.u. with the polarization oriented along the *z*-axis for (a) the benzene and (b) ethylene molecule. Green regions indicate a decrease in the electron density, while yellow regions indicate an increase. The isovalue is set to 10^–4^ a.u.

A comparison between the HF and QED-HF isotropic magnetizabilities hydrocarbons is presented in Table 1. For methane, the high degree of symmetry results in a change that is almost the same for all polarization orientations. The cavity induces different changes for acetylene when the polarization is oriented along the *x*-axis, as the σ bonds are involved. For the other two polarization orientations, the cavity effects are comparable since the π bonds are equally affected. In the case of benzene, shifts in the isotropic magnetizability can be explained by considering its aromatic character. To describe this feature, the out-of-plane component of the magnetizability tensor can be employed to examine the delocalization of the π electrons.^55^ The more delocalized the π electrons, the higher the absolute value of the out-of-plane magnetizability. As shown in Table 2, when the polarization lies along the plane of the molecule, it induces minor changes in the out-of-plane components, indicating that the π electrons exhibit a relatively small response to the quantum electromagnetic field. However, when the polarization is orthogonal to the molecular plane, decreased delocalization occurs, resulting in a decrease (in absolute value) of the isotropic magnetizability. The cavity alters the distribution of the electron density over the aromatic ring leading to a decrease in the aromatic character of the molecule. As shown in Figure 2a, the cavity field induces a contraction of the π orbitals along the *z*-axis resulting in a displacement of the electron density on the C–H bonds, and therefore a smaller electron delocalization within the molecular plane.

**Table 1:** Isotropic Magnetizabilities for Different Polarization Directions of the Cavity Field (10^–30^ J T^–2^)

molecule	HF	QED-HF, *x*	QED-HF, *y*	QED-HF, *z*
methane	–317.23	–312.68	–312.68	–312.60
ethylene	–360.39	–355.84	–359.22	–352.94
acetylene	–388.37	–382.89	–381.62	–381.62
benzene	–991.77	–990.50	–989.90	–980.39

**Table 2:** Out-of-Plane Component of the Magnetizabilities Tensors for the Benzene Molecule (10^–30^ J T^–2^)

method	χ_*zz*_
HF	–1705.46
QED-HF, *x*	–1701.75
QED-HF, *y*	–1700.82
QED-HF, *z*	–1687.65

This behavior has also been observed in the ethylene molecule, as highlighted in Figure 2b, where the largest cavity effect emerges when the polarization is aligned with the π-bond orbitals.

**Figure 3:**
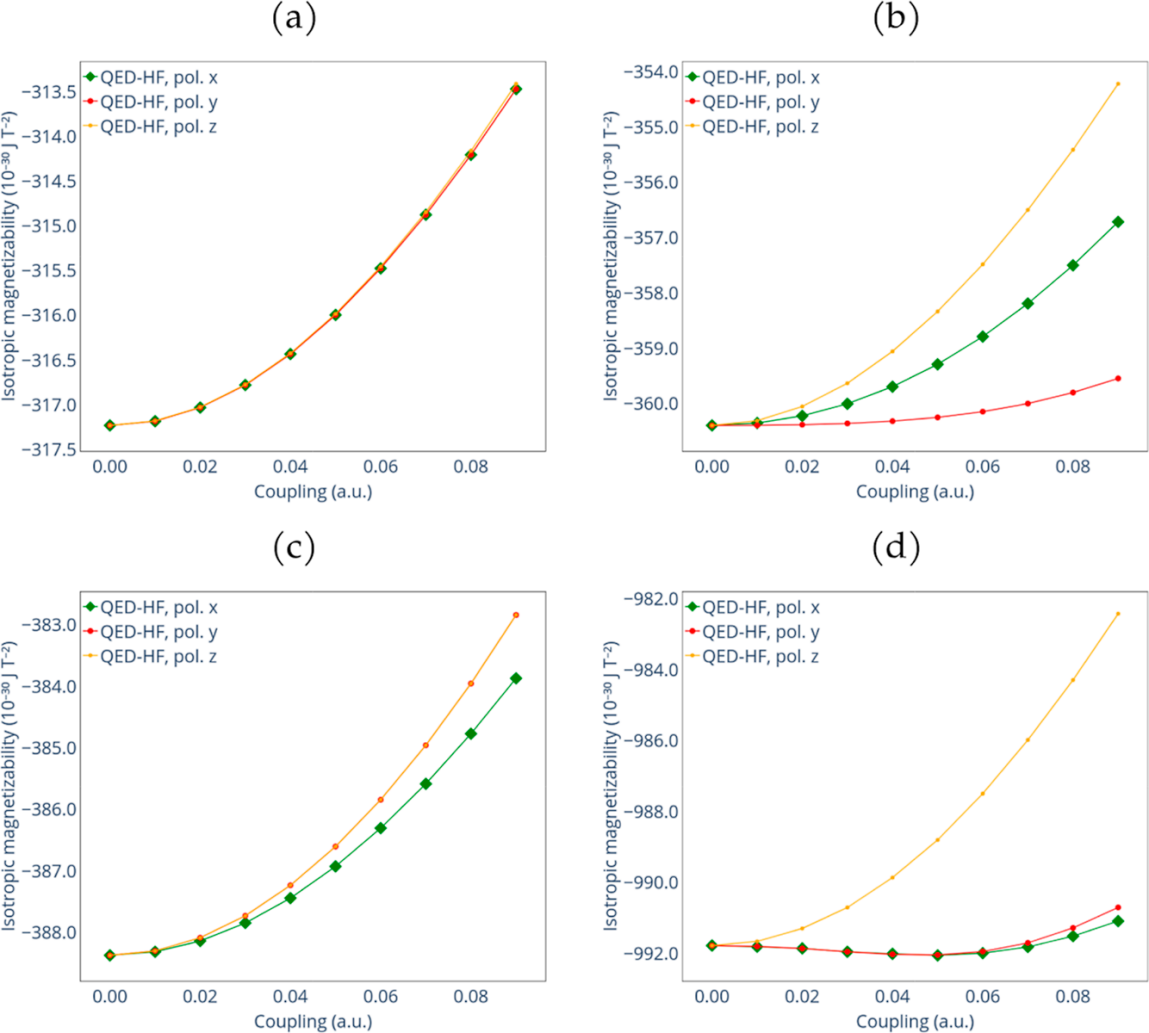
Variation in the total isotropic magnetizability at different values of coupling strength and polarization orientations for the methane (a), ethylene (b), acetylene (c), and benzene molecules (d).

In Figure 3 we illustrate the isotropic magnetizability as a function of coupling strength showing the different polarization orientation effects for each hydrocarbon. Methane shows a consistent curve shape independently of the polarization orientation (Figure 3a). Acetylene shows a similar behavior mirroring the trend observed for methane except for the polarization oriented along the bond axis (Figure 3c). However, in the case of ethylene and benzene (Figure 3b,d), the changes with the coupling confirm that the polarization orthogonal to the molecular plane produces a larger effect in the isotropic magnetizability while the coupling is increasing, affecting largely the out-of-plane component of the magnetizability. Moreover, the benzene molecule shows quite peculiar behavior when polarization is oriented along the molecular plane. Indeed, for small values of coupling strength, the magnetizability slightly increases while for larger couplings starts to decrease (in absolute value). Additionally, the in-plane polarizations affect differently the molecular orbitals, leading to different values of magnetizability when the coupling is increased. The alterations in the total isotropic magnetizabilities induced by the quantum field can be reasoned by considering the expression
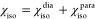
72where *χ*_iso_^dia^ and *χ*_iso_^para^ are the isotropic diamagnetic and paramagnetic contributions, respectively. The diamagnetic contribution can be evaluated as^77^
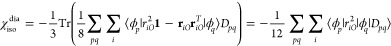
73where *D*_*pq*_ is the density matrix in the MO basis. The contribution of eq 73 to the total magnetizability is always negative and typically dominant for closed-shell molecules. As observed in the case of benzene and ethylene in Figure 2a,b, the quantum field alters the electron density by contracting it along the direction of the field polarization. Therefore, we assign the decrease (in absolute value) in the total isotropic magnetizabilities for all the investigated molecules to a decrease in the molecular diamagnetism due to the contraction of the electron density induced by the quantum field.

It is worth mentioning that the HF model effectively reproduces experimental magnetizabilities values, as the contribution of the electron correlation to this property is usually small.^84^ However, in the case of polaritons, the effects of electron-photon correlation could play a more important role on this property. Consequently, further investigations are necessary to elucidate these effects by using electron-photon correlated models, for instance, QED-CC.^44^

### 4.2 Modulation of Aromaticity

**Figure 4:**
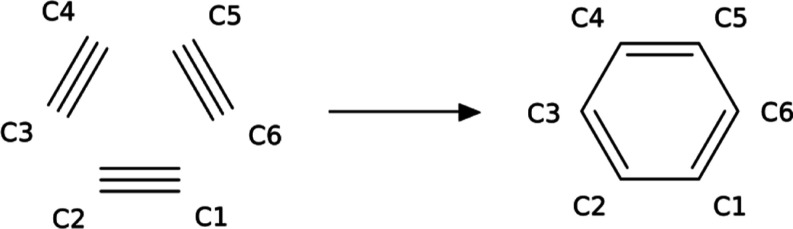
Schematic representation of the reaction mechanism for the trimerization of acetylene to benzene.

We conclude by investigating the quantum field effects on the trimerization of acetylene to benzene, represented in Figure 4. This reaction is an example of thermally allowed pericyclic reactions intensively studied in the past^58,59,85−87^ and takes place via a concerted pathway that passes through an aromatic transition state (TS). This transition state has been theoretically investigated analyzing various magnetic properties as ^1^H-NMR chemical shifts,^61,63,64^ magnetizability exaltation,^59,60,62,88^ and nucleus independent chemical shift (NICS).^58^

In this work, we employed the nucleus-independent chemical shift at the ring center, known as NICS(0), and the magnetizability exaltation, as aromaticity descriptors.^57^ We acknowledge the limitations of employing NICS(0) for computationally assessing the molecular aromaticity, as highlighted in ref (55). However, as we only aim to qualitatively analyze the effect of the quantum field on the magnetic properties exploring how the cavity can influence the aromatic transition state in the reaction pathway shown in Figure 4. The NICS(0) and the magnetizability exaltation are defined as
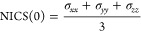
74
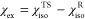
75

**Figure 5:**
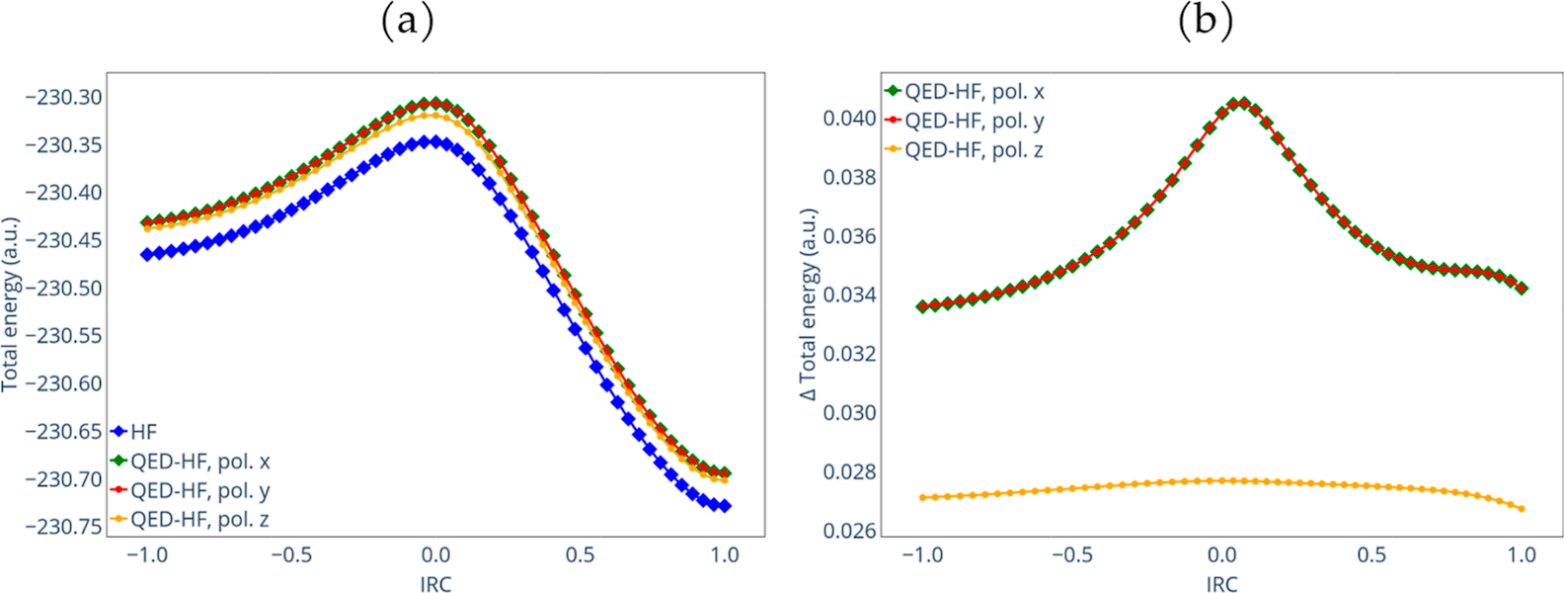
Comparison of the total energy (a) and its differences (b) for the HF and QED-HF calculations with different polarization orientations along the IRC.

In eq 74, σ_*xx*_, σ_*yy*_, and σ_*zz*_ represent the diagonal components of the nuclear shielding tensor for a ghost atom placed in the center of mass of the molecule. In eq 75, *χ*_iso_^TS^ is the isotropic magnetizability of the transition state and *χ*_iso_^R^ is the magnetizability of the reactants. As the trimerization reaction is symmetry-allowed according to the Woodward and Hoffmann rules,^89^ employing a single determinant as an electronic wave function is sufficient for qualitatively describing the essential features of the reaction. To generate the reaction pathway we employed the intrinsic reaction coordinate (IRC) calculations using the ORCA software package^80^ with the nudged elastic band and transition state optimization (NEB-TS) method.^90^ An atom-pairwise dispersion correction based on tight binding partial charges^91^ has been also applied. These calculations were performed at the DFT-B3LYP/def2-SVP level of theory. The starting geometries of the reactants and products were taken from ref (58). The reactant geometry is considered to have IRC = −1, the transition state has IRC = 0 by definition, and the equilibrium geometry of the product has IRC = 1. A set of 22 geometries has been computed from IRC = −1 to IRC = 0, and an additional 27 geometries from IRC = 0 to IRC = 1. The transition state has D_3*h*_ symmetry with a single imaginary vibrational frequency at −616.7 cm^–1^ and carbon–carbon separations of 1.23 and 2.33 Å. These findings are in line with a previous study by Jiao and Schleyer.^59^Figure 5a shows the total energies obtained for HF and QED-HF. The QED-HF calculations were carried out with λ = 0.05 a.u. for different polarization directions along the *x*-, *y*-, and *z*-axis. The reactants and products were positioned in the *xy* plane. The HF calculations replicate previous results shown in ref (59). Examining the potential energy surface along the IRC, a relatively flat region is observed from acetylene reactants to the transition state, followed by a steep descent to benzene. The QED-HF curves confirm the concerted and synchronous nature of the transition from reactants to products, even under the influence of a quantum electromagnetic field. However, Figure 5b reveals that when the polarization is orthogonal to the plane containing the reactants, a modest shift in the total energy occurs. In contrast, for the in-plane polarizations, the effect of the quantum field intensifies as the transition state is approached. This effect could be attributed to the larger polarization of the orbitals, manifested through increased oscillations of the total electronic dipole around its mean value. The activation energies in Table 3 support and confirm the observed behavior.

**Table 3:** Activation Energies (kcal mol^–1^) for the HF and QED-HF Calculations with Different Polarization Orientations

method	act. energies
HF	74.04
QED-HF, *x*	78.44
QED-HF, *y*	78.44
QED-HF, *z*	74.67

**Figure 6:**
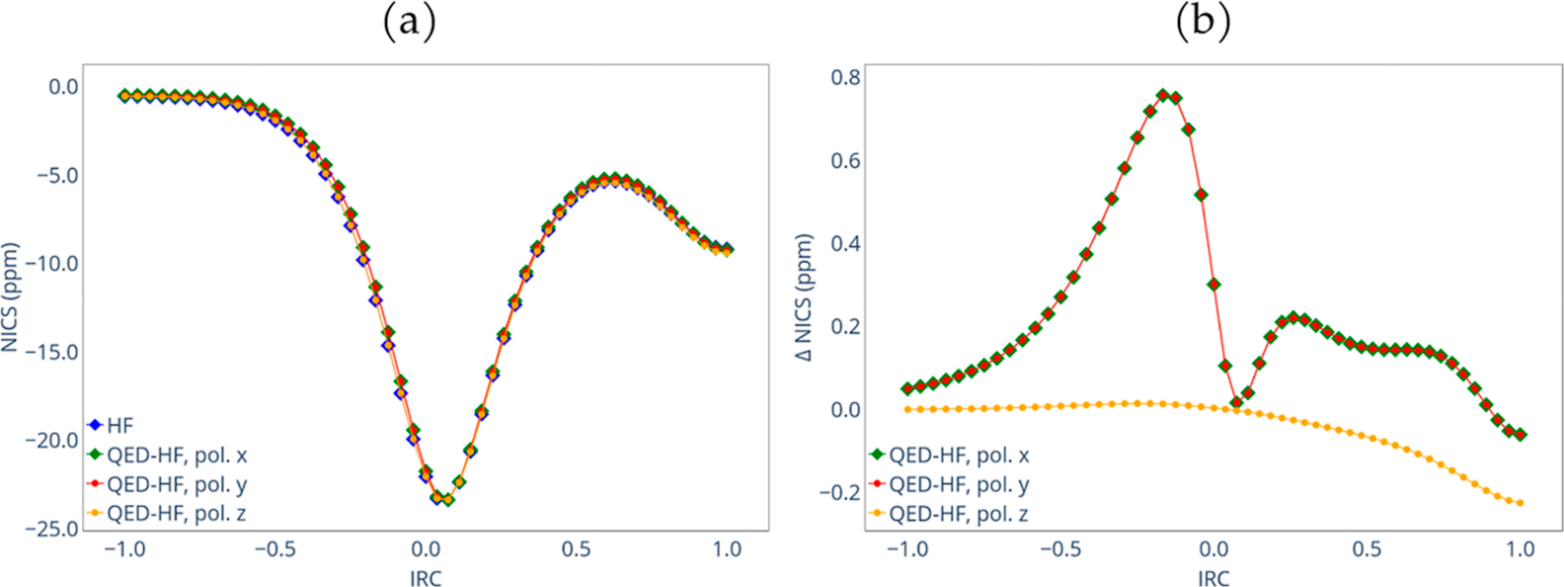
Comparison of NIC*S*_tot_(0) (a) and its differences (b) for the HF and QED-HF calculations with different polarization orientations along the IRC.

The NICS(0) results are reported in Figure 6a. The HF curve is in agreement with the findings of Havenith et al.^58^ The negative values indicate the aromatic character of the transition state and the products. As suggested by the authors, the NICS(0) remains close to zero in the early stages of the reaction, decreases to a minimum immediately after reaching the transition state, and then raises again as the paratropic character increases. Finally, it decreases again due to the formation of the π bonds. As the NICS(0) is calculated in the molecular plane, the TS NICS(0) is higher (in absolute value) due to the σ electrons ring current, which is less intense in the case of benzene. In Figure 6b, we reported the differences in the NICS(0) between the QED-HF and HF results along the IRC. When the polarization is oriented along the *z*-axis, the difference in the NICS(0) remains constant for almost all the IRC. However, at the end of the reaction path, it increases (in absolute value) meaning that the polarization of the π electrons due to the cavity increases the diatropic character. In the case of *x*- and *y*-polarization the QED-HF is lower than the HF (in absolute value) until the transition state is approached meaning that the diatropic character is decreased by the cavity. This behavior confirms that the polarizations within the molecular plane lead to a decrease in NICS(0) at the transition state. Consequently, the aromatic character is reduced by the cavity, resulting in a less stable transition state, as confirmed by the activation energy analysis. Subsequently, after reaching the transition state, the value converges toward the HF value to increase again between values ranging from 0.1 and 0.3 along the IRC, where the paratropic character decreases (in absolute value). Finally, the QED-HF approaches the HF values at the end of the reaction pathway.

In Table 4a we reported the diagonal elements of the magnetizability tensors for the transition state obtained with HF and QED-HF methods. The HF results are in line with the aromaticity evaluation of the transition state reported by Jiao et al.^59^ This agreement persists in the QED-HF results. However, for the *x*- and *y*-polarization the out-of-plane component of the tensors shows a decrease compared to HF values. This behavior is in line with the NICS(0) results, suggesting a slightly decreased aromatic character of the transition state within the cavity. Moreover, the in-plane components are almost identical due to the high degree of symmetry of the transition state. On the contrary, the polarization along the *z*-axis produces a shift in all the diagonal components of the tensor, similar to what is observed for the total energies.

**Table 4:** Diagonal Elements of the Transition State Magnetizability Tensors (a) and Magnetizability Exaltation Values (b) for the HF and QED-HF Calculations (10^–30^ J T^–2^)

(a)				(b)	
method	χ_*xx*_	χ_*yy*_	χ_*zz*_	method	χ_ex_
HF	–993.11	–993.03	–1978.91	HF	–12.80
QED-HF, *x*	–994.52	–988.02	–1830.38	QED-HF, *x*	–10.65
QED-HF, *y*	–988.10	–994.93	–1827.79	QED-HF, *y*	–10.64
QED-HF, *z*	–974.99	–974.95	–1961.37	QED-HF, *z*	–12.78

The magnetizability exaltations are shown in Table 4. The negative values are in line with the aromatic character of the transition state. Moreover, the QED-HF results confirm that *z*-polarization merely causes a shift in values, as the obtained value aligns with the HF values. On the contrary, the *x*- and *y*-directions modify the features of the transition state, decreasing its aromatic character, as demonstrated also by the analysis of NICS(0).

## 5 Conclusions

In this work, we have developed ab initio methods that explicitly include interactions with a static magnetic field and the nuclear spin degrees of freedom for molecular systems within an optical cavity. First, we introduced a minimal coupling approach that describes these interactions. Subsequently, we presented a model that includes the cavity magnetic dipole interactions with an approximate description of the quantum electromagnetic field. Finally, we further simplified this Hamiltonian by applying the dipole approximation. We developed the first implementation at the QED-HF level for calculating magnetizability and nuclear shielding tensors. The obtained results for the magnetizability of hydrocarbons indicate significant effects induced by the cavity. Indeed, the isotropic magnetizability varies depending on the polarization orientation and the coupling strength. In aromatic compounds such as benzene, we observed that the predominant effect of the cavity occurs when the polarization is orthogonal to the molecular plane. This is confirmed by changes in the out-of-plane component of the magnetizability, which indicate a decreased delocalization of the π electrons with a consequent alteration in the electron density distribution over the aromatic ring. Furthermore, we explored the effects of the optical cavity on aromaticity descriptors. The results obtained from the acetylene trimerization to benzene indicate that the cavity can modify the aromatic character of the transition state, as highlighted by NICS values and magnetizability exaltation. We demonstrated that when the polarization is oriented in the plane of molecules, there is an increase in the activation energy. This modification could lead to a shift in the equilibrium of the reaction, offering a way to govern the reaction pathway that involves aromatic transition states or intermediates, even if the effects induced by the cavity are small compared to the electron stabilization in aromatic systems. This study opens the possibility to further investigations on how molecular magnetic properties are influenced by the presence of a quantum electromagnetic field. Future analysis may include the electron–electron and electron-photon correlation in the calculation of such properties and the exploration of more reliable aromaticity descriptors, such as multidimensional NICS^92^ and global ring currents.^93^

## Data Availability

The data and the code that support the findings of this study are available from the corresponding author upon reasonable request. Examples of the input files used to run the calculations are available in ref (94).

## Supporting Information Available

The Supporting Information contains further details on the approximation introduced in Section 2.2, the geometries of the molecules, and the data shown in the graphs in Section 4.1, as well as an additional analysis of the basis set effects in the calculations presented in Section 4.1. Finally, the data shown in the graphs and the geometries of the reaction pathway presented in Section 4.2 are provided (PDF)
