# Supplementary material for: Theory of Magnetic Properties in Quantum Electrodynamics Environments: Application to Molecular Aromaticity
Source: J Chem Theory Comput. 2024 Sep 10;20(18):7841–54. doi: 10.1021/acs.jctc.4c00195 (PMC11428136; doi:10.1021/acs.jctc.4c00195)
Supplement: Supplementary file 1 — ct4c00195_si_001.pdf [file ct4c00195_si_001.pdf]

Supporting information for Theory of magnetic properties in QED environments: application to molecular aromaticity

Alberto Barlini,† Andrea Bianchi,† Enrico Ronca,*,‡ and Henrik Koch*,¶

†*Scuola Normale Superiore, Pisa, Italy*

‡*Dipartimento di Chimica, Biologia e Biotecnologie, Università degli Studi di Perugia, Perugia, Italy*

¶*Department of Chemistry, Norwegian University of Science and Technology, Trondheim, Norway*

E-mail: enrico.ronca@unipg.it; henrik.koch@ntnu.no

On the cavity field approximation

Consider the following expressions for the electric and magnetic field

$$\mathbf{E}\left(\mathbf{r}_i\right) = i\sum_{\mathbf{k}\lambda}\omega_{\mathbf{k}}\mathcal{A}_{\mathbf{k}}\boldsymbol{\epsilon}_\lambda\left(b_{\mathbf{k}\lambda} - b^\dagger_{\mathbf{k}\lambda}\right) + \frac{1}{2}\sum_{\mathbf{k}\lambda}\omega_{\mathbf{k}}\mathcal{A}_{\mathbf{k}}\left(\mathbf{r}_i \times \left(\mathbf{k}\times\boldsymbol{\epsilon}_\lambda\right)\right)\left(b_{\mathbf{k}\lambda} + b^\dagger_{\mathbf{k}\lambda}\right) \tag{1}$$

$$\mathbf{B}\left(\mathbf{r}_i\right) = i\sum_{\mathbf{k}\lambda}\mathcal{A}_{\mathbf{k}}\left(\mathbf{k}\times\boldsymbol{\epsilon}_\lambda\right)\left(b_{\mathbf{k}\lambda} - b^\dagger_{\mathbf{k}\lambda}\right). \tag{2}$$

By substituting Eq. 1 and Eq. 2 in the Maxwell's equations expressed in atomic units, we obtain

$$\nabla\cdot\mathbf{E} = 0 \tag{3}$$

$$\nabla\cdot\mathbf{B} = 0 \tag{4}$$

$$\nabla\times\mathbf{E} = -\frac{\partial\mathbf{B}}{\partial t} \tag{5}$$

$$\nabla\times\mathbf{B} \neq -\frac{1}{c^2}\frac{\partial\mathbf{E}}{\partial t}. \tag{6}$$

Here, Eq. 6 is not fulfilled. Indeed, the left-hand side and the right-hand side read as

$$\nabla \times \mathbf{B} = 0 \tag{7}$$

$$-\frac{1}{c^2}\frac{\partial \mathbf{E}}{\partial t} = \frac{1}{c^2}\sum_{\mathbf{k}\lambda}\omega_{\mathbf{k}}^2 \mathcal{A}_{\mathbf{k}} \boldsymbol{\epsilon}_\lambda \left(b_{\mathbf{k}\lambda} - b_{\mathbf{k}\lambda}^\dagger\right) + \frac{i}{2}\sum_{\mathbf{k}\lambda}\omega_{\mathbf{k}}^2 \mathcal{A}_{\mathbf{k}} \left(\mathbf{r}_i \times (\mathbf{k} \times \boldsymbol{\epsilon}_\lambda)\right)\left(b_{\mathbf{k}\lambda} - b_{\mathbf{k}\lambda}^\dagger\right), \tag{8}$$

where the time dependence of the electric field in Eq. 1 resides in the photon operators

$$b = \tilde{b}\,\text{exp}\left(i\omega t\right) \tag{9}$$

$$b^\dagger = \tilde{b}^\dagger\,\text{exp}\left(-i\omega t\right). \tag{10}$$

Although the electric field still depends on time due to the presence of photon operators, the magnetic field becomes constant in space. Thus, the electric and magnetic fields in Eq. 1 and Eq. 2 break Ampere-Maxwell's law as in the common dipole approximation.

B3LYP/def2-SVP optimized geometries

This section reports the optimized geometries in angstrom of the examined hydrocarbons used in the HF and QED-HF magnetizability.

Methane

C	0.000000	-0.000000	0.000000
H	-0.000000	-0.897870	-0.634876
H	-0.000000	0.897870	-0.634876
H	-0.897870	-0.000000	0.634876
H	0.897870	0.000000	0.634876

Ethylene

C	-0.666616	0.000006	-0.000000
C	0.666649	-0.000007	0.000001
H	-1.242769	-0.928617	0.070771
H	-1.242751	0.928640	-0.070773
H	1.242801	0.928616	-0.070771
H	1.242786	-0.928638	0.070772

Acetylene

C	-0.604660	0.000000	-0.000005
C	0.604660	0.000000	0.000005
H	-1.679229	0.000000	-0.000099
H	1.679229	0.000000	0.000099

Benzene

C	-1.216810	-0.690500	0.000008
C	-1.206456	0.708523	0.000022
C	-0.010350	-1.398981	-0.000002
C	0.010407	1.399004	0.000016
C	1.206498	-0.708489	-0.000002
C	1.216858	0.690526	0.000009
H	-2.167931	-1.230304	0.000010
H	-2.149461	1.262372	0.000031
H	-0.018509	-2.492589	-0.000015
H	0.018492	2.492614	0.000026
H	2.149504	-1.262340	-0.000005
H	2.167958	1.230365	0.000003

Modulation of magnetizabilities

This section reports the magnetizabilities data reported in the plots reported in Fig. 4. All the data are in J T^{-2} 10^{-30}.

Table 1: Isotropic magnetizabilities for the methane molecule.

QED-HF, pol. x	QED-HF, pol. y	QED-HF, pol. z
-317.228	-317.228	-317.228
-317.181	-317.181	-317.177
-317.028	-317.028	-317.024
-316.777	-316.777	-316.772
-316.431	-316.430	-316.423
-315.994	-315.994	-315.981
-315.473	-315.473	-315.454
-314.874	-314.874	-314.845
-314.204	-314.204	-314.161
-313.450	-313.470	-313.411
-312.676	-312.676	-312.601

Table 2: Isotropic magnetizabilities for the ethylene molecule.

QED-HF, pol. x	QED-HF, pol. y	QED-HF, pol. z
-360.391	-360.391	-360.391
-360.348	-360.388	-360.305
-360.218	-360.377	-360.048
-359.999	-360.354	-359.628
-359.690	-360.313	-359.052
-359.286	-360.245	-358.331
-358.785	-360.141	-357.480
-358.188	-359.995	-356.496
-357.497	-359.796	-355.408
-356.712	-359.541	-354.217
-355.840	-359.222	-352.935

Table 3: Isotropic magnetizabilities for the acetylene molecule.

QED-HF, pol. x	QED-HF, pol. y	QED-HF, pol. z
-388.371	-388.371	-388.371
-388.313	-388.299	-388.299
-388.137	-388.084	-388.084
-387.845	-387.727	-387.727
-387.439	-387.231	-387.231
-386.924	-386.601	-386.601
-386.303	-385.841	-385.841
-385.582	-384.955	-384.955
-384.767	-383.952	-383.952
-383.866	-382.837	-382.837
-382.887	-381.616	-381.616

Table 4: Isotropic magnetizabilities for the benzene molecule.

QED-HF, pol. x	QED-HF, pol. y	QED-HF, pol. z
-991.767	-991.768	-991.768
-991.801	-991.791	-991.652
-991.849	-991.851	-991.287
-991.943	-991.943	-990.696
-992.002	-992.016	-989.853
-992.046	-992.033	-988.793
-991.981	-991.937	-987.488
-991.808	-991.694	-985.975
-991.504	-991.273	-984.287
-991.079	-990.694	-982.420
-990.500	-989.900	-980.393

Further investigations on the modulation of magnetizability

In this section, we present the results for the benzene and ethylene molecules using the aug-cc-pVTZ basis set. This analysis aims to determine whether the observed behavior of the isotropic magnetizabilities, increasing more with polarization orthogonal to the molecular plane, could be attributed to the choice of basis. The following data demonstrate that the isotropic magnetizabilities consistently follow the same pattern relative to the coupling strength, as observed with the aug-cc-pVDZ basis set. Consequently, we observed minor basis effects.

Table 5: Benzene isotropic magnetizabilities for different polarization directions and coupling strength with the aug-cc-pVTZ basis set $(10^{-30}\ \mathrm{J\ T^{-2}})$.

Coupling	QED-HF, pol. x	QED-HF, pol. y	QED-HF, pol. z
0.00	-1000.328	-1000.322	-1000.321
0.01	-1000.336	-1000.332	-1000.232
0.02	-1000.357	-1000.35	-999.956
0.03	-1000.367	-1000.368	-999.469
0.04	-1000.336	-1000.344	-998.758
0.05	-1000.269	-1000.27	-997.799
0.06	-1000.119	-1000.093	-996.568
0.07	-999.897	-999.829	-995.037
0.08	-999.549	-999.432	-993.212
0.09	-999.126	-998.91	-991.055
0.10	-998.549	-998.22	-988.551

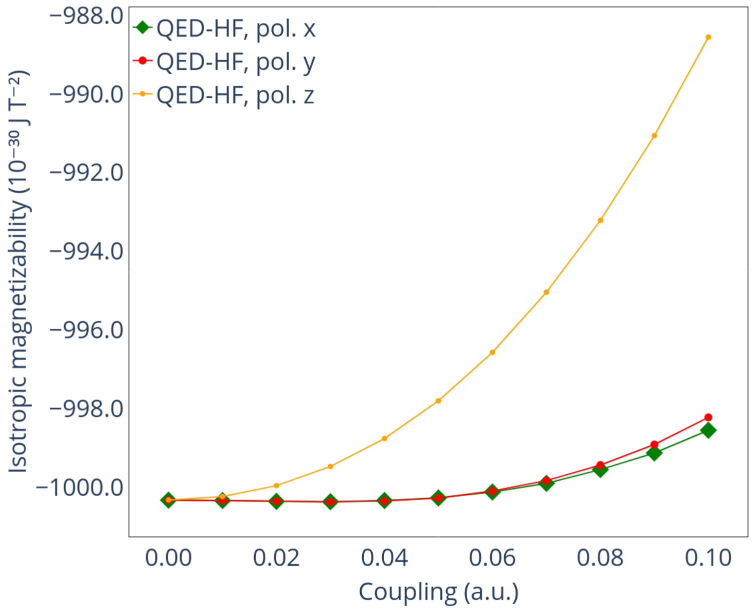

Figure 1: Variations in the total isotropic magnetizabilities of benzene for different polarization directions and coupling strength with the aug-cc-pVTZ basis set $(10^{-30}\ \mathrm{J\ T^{-2}})$.

Table 6: Ethylene isotropic magnetizabilities for different polarization directions and coupling strength with the aug-cc-pVTZ basis set $(10^{-30}\ \mathrm{J\ T^{-2}})$.

Coupling	QED-HF, pol. x	QED-HF, pol. y	QED-HF, pol. z
0.00	-357.179	-357.179	-357.179
0.01	-357.146	-357.159	-357.077
0.02	-357.048	-357.099	-356.775
0.03	-356.885	-356.995	-356.28
0.04	-356.659	-356.845	-355.604
0.05	-356.373	-356.644	-354.762
0.06	-356.028	-356.39	-353.77
0.07	-355.63	-356.078	-352.64
0.08	-355.185	-355.709	-351.384
0.09	-354.699	-355.282	-350.01
0.10	-354.179	-354.801	-348.523

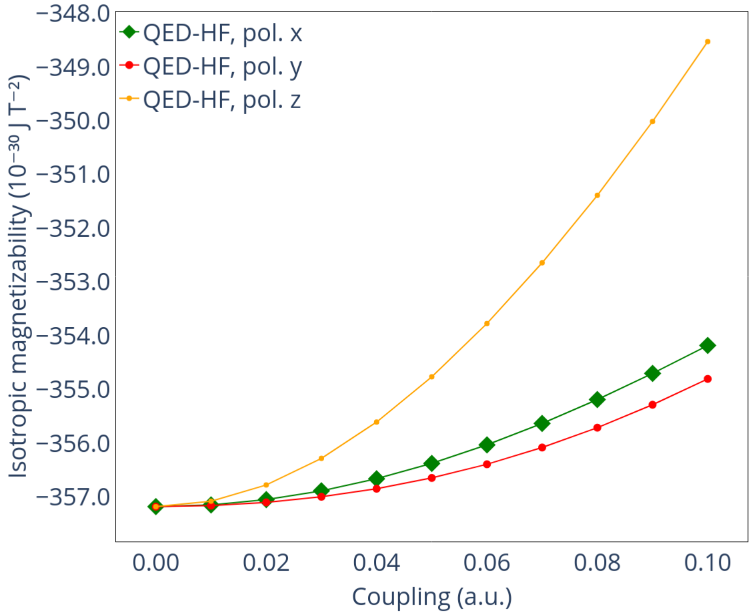

Figure 2: Variations in the total isotropic magnetizabilities of ethylene for different polarization directions and coupling strength with the aug-cc-pVTZ basis set $(10^{-30}$ J T$^{-2})$.

Modulation of aromaticity

This section reports the data reported in the plots of the total energies and NICS(0) along the trimerization reaction pathway. The energies are in atomic units and the NICS(0) are in ppm.

HF	QED-HF, pol. x	QED-HF, pol. y	QED-HF, pol. z
-230.465	-230.431	-230.431	-230.438
-230.461	-230.427	-230.427	-230.434
-230.458	-230.423	-230.423	-230.430
-230.455	-230.421	-230.421	-230.428
-230.452	-230.418	-230.418	-230.425
-230.449	-230.415	-230.415	-230.422
-230.445	-230.411	-230.411	-230.418
-230.441	-230.406	-230.406	-230.414
-230.436	-230.401	-230.401	-230.409
-230.431	-230.396	-230.396	-230.403
-230.425	-230.390	-230.390	-230.398
-230.420	-230.385	-230.385	-230.392
-230.414	-230.379	-230.379	-230.386
-230.408	-230.372	-230.372	-230.380
-230.401	-230.366	-230.366	-230.374
-230.394	-230.358	-230.358	-230.367
-230.387	-230.351	-230.351	-230.360
-230.380	-230.344	-230.344	-230.353
-230.373	-230.336	-230.336	-230.345
-230.366	-230.329	-230.329	-230.339
-230.360	-230.321	-230.322	-230.332
-230.354	-230.315	-230.315	-230.326
-230.350	-230.310	-230.310	-230.322
-230.348	-230.308	-230.308	-230.321
-230.350	-230.309	-230.309	-230.322
-230.357	-230.316	-230.316	-230.329
-230.370	-230.329	-230.329	-230.342
-230.394	-230.354	-230.354	-230.366
-230.446	-230.407	-230.407	-230.417

Continue to the next page.

HF	QED-HF, pol. x	QED-HF, pol. y	QED-HF, pol. z
-230.491	-230.453	-230.453	-230.463
-230.571	-230.534	-230.534	-230.543
-230.627	-230.590	-230.590	-230.599
-230.673	-230.636	-230.636	-230.645
-230.697	-230.661	-230.661	-230.669
-230.724	-230.689	-230.689	-230.697
-230.728	-230.693	-230.693	-230.701
-230.728	-230.694	-230.694	-230.701
-230.726	-230.691	-230.691	-230.699
-230.726	-230.691	-230.691	-230.699
-230.727	-230.692	-230.692	-230.700
-230.728	-230.693	-230.693	-230.701
-230.728	-230.693	-230.693	-230.701
-230.727	-230.692	-230.692	-230.700
-230.726	-230.691	-230.691	-230.699
-230.726	-230.691	-230.691	-230.699
-230.727	-230.692	-230.692	-230.700
-230.726	-230.692	-230.692	-230.699
-230.728	-230.694	-230.694	-230.701

HF	QED-HF, pol. x	QED-HF, pol. y	QED-HF, pol. z
-0.536	-0.486	-0.486	-0.536
-0.544	-0.488	-0.488	-0.544
-0.556	-0.493	-0.493	-0.555
-0.574	-0.503	-0.503	-0.573
-0.601	-0.520	-0.520	-0.599
-0.640	-0.547	-0.547	-0.638
-0.697	-0.590	-0.590	-0.694
-0.777	-0.654	-0.653	-0.774
-0.890	-0.746	-0.746	-0.885
-1.044	-0.877	-0.876	-1.039
-1.255	-1.059	-1.058	-1.249
-1.538	-1.307	-1.307	-1.531
-1.914	-1.643	-1.642	-1.905
-2.408	-2.089	-2.088	-2.398
-3.050	-2.676	-2.675	-3.039
-3.876	-3.439	-3.438	-3.863
-4.924	-4.417	-4.416	-4.911
-6.237	-5.655	-5.654	-6.222
-7.852	-7.197	-7.196	-7.837
-9.797	-9.079	-9.077	-9.782
-12.072	-11.315	-11.314	-12.058
-14.627	-13.877	-13.875	-14.615
-17.327	-16.653	-16.651	-17.317
-19.923	-19.405	-19.404	-19.916
-22.042	-21.740	-21.739	-22.038
-23.272	-23.167	-23.166	-23.271
-23.358	-23.342	-23.342	-23.360
-22.367	-22.326	-22.327	-22.372
-20.624	-20.512	-20.513	-20.634
-18.508	-18.333	-18.333	-18.522
-16.310	-16.099	-16.100	-16.330
-14.215	-13.994	-13.994	-14.240
-12.320	-12.104	-12.104	-12.351
-10.665	-10.462	-10.463	-10.702

Continue to the next page.

HF	QED-HF, pol. x	QED-HF, pol. y	QED-HF, pol. z
-9.260	-9.073	-9.073	-9.302
-8.096	-7.924	-7.925	-8.145
-7.159	-7.000	-7.000	-7.215
-6.433	-6.281	-6.282	-6.495
-5.900	-5.754	-5.754	-5.969
-5.548	-5.403	-5.403	-5.625
-5.364	-5.220	-5.220	-5.450
-5.339	-5.194	-5.194	-5.435
-5.463	-5.320	-5.320	-5.570
-5.726	-5.587	-5.587	-5.845
-6.113	-5.984	-5.984	-6.245
-6.603	-6.491	-6.491	-6.749
-7.163	-7.078	-7.078	-7.326
-7.751	-7.700	-7.700	-7.930
-8.310	-8.298	-8.298	-8.504
-8.773	-8.798	-8.799	-8.982
-9.078	-9.129	-9.129	-9.296
-9.173	-9.233	-9.233	-9.398

NEB-TS optimized geometries

This section reports the optimized geometries in angstrom of the trimerization reaction pathway. The first of the following geometries corresponds to the transition state geometry.

Transition state geometry

C	1.699255	0.616421	0.000000
C	1.699255	-0.616421	0.000000
C	-0.315404	-1.780419	-0.000000
C	-0.315403	1.780419	0.000000
C	-1.383900	-1.165609	0.000000
C	-1.383900	1.165609	-0.000000
H	2.160072	-1.586595	0.000000
H	2.160072	1.586595	-0.000000
H	0.294821	2.664332	0.000000
H	0.294821	-2.664333	-0.000000
H	-2.454316	1.077612	-0.000000
H	-2.454316	-1.077612	0.000000

Reaction patways geometries

C	2.141858	0.592962	0.000000
C	2.141858	-0.592962	0.000000
C	-0.557895	-2.154007	-0.000000
C	-0.557895	2.154007	-0.000000
C	-1.583800	-1.561192	-0.000000
C	-1.583800	1.561192	-0.000000
H	2.194181	-1.645536	0.000000
H	2.194181	1.645536	0.000000
H	0.323820	2.723740	0.000000
H	0.323820	-2.723740	0.000000
H	-2.519930	1.080194	-0.000000
H	-2.519930	-1.080194	-0.000000

C	2.121582	0.593797	0.000000
C	2.121582	-0.593797	0.000000
C	-0.547026	-2.136804	-0.000000
C	-0.547026	2.136804	-0.000000
C	-1.574396	-1.543155	-0.000000
C	-1.574396	1.543155	-0.000000
H	2.194029	-1.644578	0.000000
H	2.194029	1.644578	0.000000
H	0.323149	2.723100	0.000000
H	0.323149	-2.723100	0.000000
H	-2.519066	1.080463	-0.000000
H	-2.519066	-1.080463	-0.000000

C	2.101311	0.594709	0.000000
C	2.101311	-0.594709	0.000000
C	-0.536093	-2.119645	-0.000000
C	-0.536093	2.119645	-0.000000
C	-1.565062	-1.525085	-0.000000
C	-1.565062	1.525085	-0.000000
H	2.194095	-1.643372	0.000000
H	2.194095	1.643372	0.000000
H	0.322149	2.722526	0.000000
H	0.322149	-2.722526	0.000000
H	-2.518098	1.081048	-0.000000
H	-2.518098	-1.081048	-0.000000

C	2.081044	0.595693	0.000000
C	2.081044	-0.595693	0.000000
C	-0.525099	-2.102525	-0.000000
C	-0.525099	2.102525	-0.000000
C	-1.555791	-1.506983	-0.000000
C	-1.555791	1.506983	-0.000000
H	2.194357	-1.641897	0.000000
H	2.194357	1.641897	0.000000
H	0.320815	2.721987	0.000000
H	0.320815	-2.721987	0.000000
H	-2.516994	1.081940	-0.000000
H	-2.516994	-1.081940	-0.000000

C	2.060777	0.596747	0.000000
C	2.060777	-0.596747	0.000000
C	-0.514046	-2.085442	-0.000000
C	-0.514046	2.085442	-0.000000
C	-1.546581	-1.488848	-0.000000
C	-1.546581	1.488848	-0.000000
H	2.194792	-1.640136	0.000000
H	2.194792	1.640136	0.000000
H	0.319143	2.721455	0.000000
H	0.319143	-2.721455	0.000000
H	-2.515728	1.083128	-0.000000
H	-2.515728	-1.083128	-0.000000

C	2.040510	0.597868	0.000000
C	2.040510	-0.597868	0.000000
C	-0.502935	-2.068392	-0.000000
C	-0.502935	2.068392	-0.000000
C	-1.537428	-1.470679	-0.000000
C	-1.537428	1.470679	-0.000000
H	2.195379	-1.638071	0.000000
H	2.195379	1.638071	0.000000
H	0.317128	2.720903	0.000000
H	0.317128	-2.720903	0.000000
H	-2.514274	1.084601	-0.000000
H	-2.514274	-1.084601	-0.000000

C	2.020240	0.599052	0.000000
C	2.020240	-0.599052	0.000000
C	-0.491767	-2.051372	-0.000000
C	-0.491767	2.051372	-0.000000
C	-1.528328	-1.452477	-0.000000
C	-1.528328	1.452477	-0.000000
H	2.196097	-1.635685	0.000000
H	2.196097	1.635685	0.000000
H	0.314768	2.720303	0.000000
H	0.314768	-2.720303	0.000000
H	-2.512606	1.086351	-0.000000
H	-2.512606	-1.086351	-0.000000

C	1.999967	0.600298	0.000000
C	1.999967	-0.600298	0.000000
C	-0.480544	-2.034380	-0.000000
C	-0.480544	2.034380	-0.000000
C	-1.519279	-1.434241	-0.000000
C	-1.519279	1.434241	-0.000000
H	2.196926	-1.632964	0.000000
H	2.196926	1.632964	0.000000
H	0.312060	2.719630	0.000000
H	0.312060	-2.719630	0.000000
H	-2.510702	1.088365	-0.000000
H	-2.510702	-1.088365	-0.000000

C	1.979692	0.601603	0.000000
C	1.979692	-0.601603	0.000000
C	-0.469270	-2.017417	-0.000000
C	-0.469270	2.017417	-0.000000
C	-1.510281	-1.415974	-0.000000
C	-1.510281	1.415974	-0.000000
H	2.197844	-1.629894	0.000000
H	2.197844	1.629894	0.000000
H	0.309003	2.718861	0.000000
H	0.309003	-2.718861	0.000000
H	-2.508542	1.090634	-0.000000
H	-2.508542	-1.090634	-0.000000

C	1.959418	0.602965	0.000000
C	1.959418	-0.602965	0.000000
C	-0.457947	-2.000485	-0.000000
C	-0.457947	2.000485	-0.000000
C	-1.501331	-1.397680	-0.000000
C	-1.501331	1.397680	-0.000000
H	2.198835	-1.626463	0.000000
H	2.198835	1.626463	0.000000
H	0.305597	2.717972	0.000000
H	0.305597	-2.717972	0.000000
H	-2.506102	1.093145	-0.000000
H	-2.506102	-1.093145	-0.000000

C	1.939150	0.604382	0.000000
C	1.939150	-0.604382	0.000000
C	-0.446581	-1.983584	-0.000000
C	-0.446581	1.983584	-0.000000
C	-1.492431	-1.379363	-0.000000
C	-1.492431	1.379363	-0.000000
H	2.199876	-1.622662	0.000000
H	2.199876	1.622662	0.000000
H	0.301842	2.716941	0.000000
H	0.301842	-2.716941	0.000000
H	-2.503367	1.095888	-0.000000
H	-2.503367	-1.095888	-0.000000

C	1.918893	0.605851	0.000000
C	1.918893	-0.605851	0.000000
C	-0.435175	-1.966721	-0.000000
C	-0.435175	1.966721	-0.000000
C	-1.483582	-1.361030	-0.000000
C	-1.483582	1.361030	-0.000000
H	2.200951	-1.618481	0.000000
H	2.200951	1.618481	0.000000
H	0.297742	2.715748	0.000000
H	0.297742	-2.715748	0.000000
H	-2.500318	1.098850	-0.000000
H	-2.500318	-1.098850	-0.000000

C	1.898654	0.607370	0.000000
C	1.898654	-0.607370	0.000000
C	-0.423735	-1.949900	-0.000000
C	-0.423735	1.949900	-0.000000
C	-1.474786	-1.342688	-0.000000
C	-1.474786	1.342688	-0.000000
H	2.202041	-1.613913	0.000000
H	2.202041	1.613913	0.000000
H	0.293298	2.714373	0.000000
H	0.293298	-2.714373	0.000000
H	-2.496942	1.102019	-0.000000
H	-2.496942	-1.102019	-0.000000

C	1.878443	0.608937	0.000000
C	1.878443	-0.608937	0.000000
C	-0.412268	-1.933126	-0.000000
C	-0.412268	1.933126	-0.000000
C	-1.466044	-1.324346	-0.000000
C	-1.466044	1.324346	-0.000000
H	2.203129	-1.608953	0.000000
H	2.203129	1.608953	0.000000
H	0.288517	2.712799	0.000000
H	0.288517	-2.712799	0.000000
H	-2.493223	1.105382	-0.000000
H	-2.493223	-1.105382	-0.000000

C	1.858269	0.610550	0.000000
C	1.858269	-0.610550	0.000000
C	-0.400779	-1.916409	-0.000000
C	-0.400779	1.916409	-0.000000
C	-1.457360	-1.306012	-0.000000
C	-1.457360	1.306012	-0.000000
H	2.204197	-1.603598	0.000000
H	2.204197	1.603598	0.000000
H	0.283403	2.711010	0.000000
H	0.283403	-2.711010	0.000000
H	-2.489151	1.108926	-0.000000
H	-2.489151	-1.108926	-0.000000

C	1.838142	0.612207	0.000000
C	1.838142	-0.612207	0.000000
C	-0.389276	-1.899754	-0.000000
C	-0.389276	1.899754	-0.000000
C	-1.448737	-1.287696	-0.000000
C	-1.448737	1.287696	-0.000000
H	2.205229	-1.597846	0.000000
H	2.205229	1.597846	0.000000
H	0.277965	2.708989	0.000000
H	0.277965	-2.708989	0.000000
H	-2.484716	1.112636	-0.000000
H	-2.484716	-1.112636	-0.000000

C	1.818072	0.613906	0.000000
C	1.818072	-0.613906	0.000000
C	-0.377767	-1.883171	-0.000000
C	-0.377767	1.883171	-0.000000
C	-1.440180	-1.269410	-0.000000
C	-1.440180	1.269410	-0.000000
H	2.206208	-1.591699	0.000000
H	2.206208	1.591699	0.000000
H	0.272211	2.706724	0.000000
H	0.272211	-2.706724	0.000000
H	-2.479912	1.116499	-0.000000
H	-2.479912	-1.116499	-0.000000

C	1.798073	0.615644	0.000000
C	1.798073	-0.615644	0.000000
C	-0.366259	-1.866669	-0.000000
C	-0.366259	1.866669	-0.000000
C	-1.431691	-1.251164	-0.000000
C	-1.431691	1.251164	-0.000000
H	2.207119	-1.585158	0.000000
H	2.207119	1.585158	0.000000
H	0.266152	2.704202	0.000000
H	0.266152	-2.704202	0.000000
H	-2.474732	1.120499	-0.000000
H	-2.474732	-1.120499	-0.000000

C	1.778157	0.617420	0.000000
C	1.778157	-0.617420	0.000000
C	-0.354760	-1.850257	-0.000000
C	-0.354760	1.850257	-0.000000
C	-1.423276	-1.232970	-0.000000
C	-1.423276	1.232970	-0.000000
H	2.207948	-1.578229	0.000000
H	2.207948	1.578229	0.000000
H	0.259800	2.701413	0.000000
H	0.259800	-2.701413	0.000000
H	-2.469172	1.124621	-0.000000
H	-2.469172	-1.124621	-0.000000

C	1.758336	0.619232	0.000000
C	1.758336	-0.619232	0.000000
C	-0.343279	-1.833947	-0.000000
C	-0.343279	1.833947	-0.000000
C	-1.414940	-1.214842	-0.000000
C	-1.414940	1.214842	-0.000000
H	2.208678	-1.570920	0.000000
H	2.208678	1.570920	0.000000
H	0.253169	2.698348	0.000000
H	0.253169	-2.698348	0.000000
H	-2.463235	1.128847	-0.000000
H	-2.463235	-1.128847	-0.000000

C	1.738630	0.621077	0.000000
C	1.738630	-0.621077	0.000000
C	-0.331827	-1.817753	-0.000000
C	-0.331827	1.817753	-0.000000
C	-1.406690	-1.196795	-0.000000
C	-1.406690	1.196795	-0.000000
H	2.209296	-1.563242	0.000000
H	2.209296	1.563242	0.000000
H	0.246276	2.695001	0.000000
H	0.246276	-2.695001	0.000000
H	-2.456921	1.133161	-0.000000
H	-2.456921	-1.133161	-0.000000

C	1.719064	0.622953	0.000000
C	1.719064	-0.622953	0.000000
C	-0.320417	-1.801695	-0.000000
C	-0.320417	1.801695	-0.000000
C	-1.398536	-1.178854	-0.000000
C	-1.398536	1.178854	-0.000000
H	2.209790	-1.555213	0.000000
H	2.209790	1.555213	0.000000
H	0.239145	2.691369	0.000000
H	0.239145	-2.691369	0.000000
H	-2.450240	1.137541	-0.000000
H	-2.450240	-1.137541	-0.000000

C	1.699662	0.624859	0.000000
C	1.699662	-0.624859	0.000000
C	-0.309066	-1.785795	-0.000000
C	-0.309066	1.785795	-0.000000
C	-1.390491	-1.161040	-0.000000
C	-1.390491	1.161040	-0.000000
H	2.210145	-1.546856	0.000000
H	2.210145	1.546856	0.000000
H	0.231799	2.687454	0.000000
H	0.231799	-2.687454	0.000000
H	-2.443206	1.141964	-0.000000
H	-2.443206	-1.141964	-0.000000

C	1.680442	0.626795	0.000000
C	1.680442	-0.626795	0.000000
C	-0.297778	-1.770067	-0.000000
C	-0.297778	1.770067	-0.000000
C	-1.382562	-1.143368	-0.000000
C	-1.382562	1.143368	-0.000000
H	2.210351	-1.538194	0.000000
H	2.210351	1.538194	0.000000
H	0.224268	2.683256	0.000000
H	0.224268	-2.683256	0.000000
H	-2.435831	1.146410	-0.000000
H	-2.435831	-1.146410	-0.000000

C	1.661397	0.628759	0.000000
C	1.661397	-0.628759	0.000000
C	-0.286554	-1.754504	-0.000000
C	-0.286554	1.754504	-0.000000
C	-1.374744	-1.125833	-0.000000
C	-1.374744	1.125833	-0.000000
H	2.210396	-1.529237	0.000000
H	2.210396	1.529237	0.000000
H	0.216563	2.678772	0.000000
H	0.216563	-2.678772	0.000000
H	-2.428121	1.150864	-0.000000
H	-2.428121	-1.150864	-0.000000

C	1.642500	0.630751	0.000000
C	1.642500	-0.630751	0.000000
C	-0.275382	-1.739084	-0.000000
C	-0.275382	1.739084	-0.000000
C	-1.367026	-1.108413	-0.000000
C	-1.367026	1.108413	-0.000000
H	2.210272	-1.519982	0.000000
H	2.210272	1.519982	0.000000
H	0.208685	2.673994	0.000000
H	0.208685	-2.673994	0.000000
H	-2.420068	1.155320	-0.000000
H	-2.420068	-1.155320	-0.000000

C	1.623733	0.632768	0.000000
C	1.623733	-0.632768	0.000000
C	-0.264251	-1.723787	-0.000000
C	-0.264251	1.723787	-0.000000
C	-1.359394	-1.091092	-0.000000
C	-1.359394	1.091092	-0.000000
H	2.209974	-1.510423	0.000000
H	2.209974	1.510423	0.000000
H	0.200636	2.668911	0.000000
H	0.200636	-2.668911	0.000000
H	-2.411664	1.159775	-0.000000
H	-2.411664	-1.159775	-0.000000

C	1.605091	0.634810	0.000000
C	1.605091	-0.634810	0.000000
C	-0.253161	-1.708610	-0.000000
C	-0.253161	1.708610	-0.000000
C	-1.351846	-1.073866	-0.000000
C	-1.351846	1.073866	-0.000000
H	2.209492	-1.500572	0.000000
H	2.209492	1.500572	0.000000
H	0.192427	2.663523	0.000000
H	0.192427	-2.663523	0.000000
H	-2.402915	1.164216	-0.000000
H	-2.402915	-1.164216	-0.000000

C	1.586584	0.636878	0.000000
C	1.586584	-0.636878	0.000000
C	-0.242117	-1.693563	-0.000000
C	-0.242117	1.693563	-0.000000
C	-1.344388	-1.056745	-0.000000
C	-1.344388	1.056745	-0.000000
H	2.208818	-1.490452	0.000000
H	2.208818	1.490452	0.000000
H	0.184083	2.657836	0.000000
H	0.184083	-2.657836	0.000000
H	-2.393838	1.168625	-0.000000
H	-2.393838	-1.168625	-0.000000

C	1.568217	0.638969	0.000000
C	1.568217	-0.638969	0.000000
C	-0.231124	-1.678648	-0.000000
C	-0.231124	1.678648	-0.000000
C	-1.337021	-1.039733	-0.000000
C	-1.337021	1.039733	-0.000000
H	2.207943	-1.480081	0.000000
H	2.207943	1.480081	0.000000
H	0.175624	2.651849	0.000000
H	0.175624	-2.651849	0.000000
H	-2.384443	1.172985	-0.000000
H	-2.384443	-1.172985	-0.000000

C	1.549995	0.641087	0.000000
C	1.549995	-0.641087	0.000000
C	-0.220179	-1.663870	-0.000000
C	-0.220179	1.663870	-0.000000
C	-1.329749	-1.022832	-0.000000
C	-1.329749	1.022832	-0.000000
H	2.206853	-1.469471	0.000000
H	2.206853	1.469471	0.000000
H	0.167068	2.645556	0.000000
H	0.167068	-2.645556	0.000000
H	-2.374732	1.177277	-0.000000
H	-2.374732	-1.177277	-0.000000

C	1.531942	0.643230	0.000000
C	1.531942	-0.643230	0.000000
C	-0.209297	-1.649250	-0.000000
C	-0.209297	1.649250	-0.000000
C	-1.322583	-1.006065	-0.000000
C	-1.322583	1.006065	-0.000000
H	2.205547	-1.458663	0.000000
H	2.205547	1.458663	0.000000
H	0.158450	2.638979	0.000000
H	0.158450	-2.638979	0.000000
H	-2.364743	1.181481	-0.000000
H	-2.364743	-1.181481	-0.000000

C	1.514062	0.645399	0.000000
C	1.514062	-0.645399	0.000000
C	-0.198480	-1.634792	-0.000000
C	-0.198480	1.634792	-0.000000
C	-1.315526	-0.989436	-0.000000
C	-1.315526	0.989436	-0.000000
H	2.204020	-1.447680	0.000000
H	2.204020	1.447680	0.000000
H	0.149792	2.632124	0.000000
H	0.149792	-2.632124	0.000000
H	-2.354492	1.185580	-0.000000
H	-2.354492	-1.185580	-0.000000

C	1.496356	0.647592	0.000000
C	1.496356	-0.647592	0.000000
C	-0.187726	-1.620495	-0.000000
C	-0.187726	1.620495	-0.000000
C	-1.308578	-0.972944	-0.000000
C	-1.308578	0.972944	-0.000000
H	2.202269	-1.436542	0.000000
H	2.202269	1.436542	0.000000
H	0.141115	2.624999	0.000000
H	0.141115	-2.624999	0.000000
H	-2.343995	1.189562	-0.000000
H	-2.343995	-1.189562	-0.000000

C	1.478821	0.649812	0.000000
C	1.478821	-0.649812	0.000000
C	-0.177037	-1.606359	-0.000000
C	-0.177037	1.606359	-0.000000
C	-1.301738	-0.956587	-0.000000
C	-1.301738	0.956587	-0.000000
H	2.200289	-1.425271	0.000000
H	2.200289	1.425271	0.000000
H	0.132436	2.617610	0.000000
H	0.132436	-2.617610	0.000000
H	-2.333269	1.193413	-0.000000
H	-2.333269	-1.193413	-0.000000

C	1.461454	0.652060	0.000000
C	1.461454	-0.652060	0.000000
C	-0.166407	-1.592381	-0.000000
C	-0.166407	1.592381	-0.000000
C	-1.295006	-0.940363	-0.000000
C	-1.295006	0.940363	-0.000000
H	2.198079	-1.413885	0.000000
H	2.198079	1.413885	0.000000
H	0.123776	2.609965	0.000000
H	0.123776	-2.609965	0.000000
H	-2.322328	1.197120	-0.000000
H	-2.322328	-1.197120	-0.000000

C	1.444251	0.654336	0.000000
C	1.444251	-0.654336	0.000000
C	-0.155835	-1.578558	-0.000000
C	-0.155835	1.578558	-0.000000
C	-1.288382	-0.924266	-0.000000
C	-1.288382	0.924266	-0.000000
H	2.195634	-1.402406	0.000000
H	2.195634	1.402406	0.000000
H	0.115150	2.602073	0.000000
H	0.115150	-2.602073	0.000000
H	-2.311190	1.200672	-0.000000
H	-2.311190	-1.200672	-0.000000

C	1.427207	0.656642	0.000000
C	1.427207	-0.656642	0.000000
C	-0.145316	-1.564887	-0.000000
C	-0.145316	1.564887	-0.000000
C	-1.281864	-0.908293	-0.000000
C	-1.281864	0.908293	-0.000000
H	2.192954	-1.390850	0.000000
H	2.192954	1.390850	0.000000
H	0.106577	2.593940	0.000000
H	0.106577	-2.593940	0.000000
H	-2.299869	1.204058	-0.000000
H	-2.299869	-1.204058	-0.000000

C	1.410316	0.658980	0.000000
C	1.410316	-0.658980	0.000000
C	-0.134846	-1.551362	-0.000000
C	-0.134846	1.551362	-0.000000
C	-1.275449	-0.892436	-0.000000
C	-1.275449	0.892436	-0.000000
H	2.190039	-1.379237	0.000000
H	2.190039	1.379237	0.000000
H	0.098073	2.585576	0.000000
H	0.098073	-2.585576	0.000000
H	-2.288380	1.207269	-0.000000
H	-2.288380	-1.207269	-0.000000

C	1.393572	0.661351	0.000000
C	1.393572	-0.661351	0.000000
C	-0.124419	-1.537981	-0.000000
C	-0.124419	1.537981	-0.000000
C	-1.269136	-0.876689	-0.000000
C	-1.269136	0.876689	-0.000000
H	2.186887	-1.367585	0.000000
H	2.186887	1.367585	0.000000
H	0.089653	2.576988	0.000000
H	0.089653	-2.576988	0.000000
H	-2.276738	1.210295	-0.000000
H	-2.276738	-1.210295	-0.000000

C	1.376966	0.663758	0.000000
C	1.376966	-0.663758	0.000000
C	-0.114031	-1.524735	-0.000000
C	-0.114031	1.524735	-0.000000
C	-1.262924	-0.861044	-0.000000
C	-1.262924	0.861044	-0.000000
H	2.183498	-1.355909	0.000000
H	2.183498	1.355909	0.000000
H	0.081330	2.568186	0.000000
H	0.081330	-2.568186	0.000000
H	-2.264959	1.213127	-0.000000
H	-2.264959	-1.213127	-0.000000

C	1.360491	0.666203	0.000000
C	1.360491	-0.666203	0.000000
C	-0.103676	-1.511621	-0.000000
C	-0.103676	1.511621	-0.000000
C	-1.256810	-0.845494	-0.000000
C	-1.256810	0.845494	-0.000000
H	2.179873	-1.344227	0.000000
H	2.179873	1.344227	0.000000
H	0.073119	2.559178	0.000000
H	0.073119	-2.559178	0.000000
H	-2.253057	1.215759	-0.000000
H	-2.253057	-1.215759	-0.000000

C	1.344137	0.668688	0.000000
C	1.344137	-0.668688	0.000000
C	-0.093347	-1.498631	-0.000000
C	-0.093347	1.498631	-0.000000
C	-1.250791	-0.830030	-0.000000
C	-1.250791	0.830030	-0.000000
H	2.176013	-1.332553	0.000000
H	2.176013	1.332553	0.000000
H	0.065032	2.549972	0.000000
H	0.065032	-2.549972	0.000000
H	-2.241044	1.218183	-0.000000
H	-2.241044	-1.218183	-0.000000

C	1.327896	0.671216	0.000000
C	1.327896	-0.671216	0.000000
C	-0.083036	-1.485759	-0.000000
C	-0.083036	1.485759	-0.000000
C	-1.244865	-0.814641	-0.000000
C	-1.244865	0.814641	-0.000000
H	2.171919	-1.320902	0.000000
H	2.171919	1.320902	0.000000
H	0.057081	2.540577	0.000000
H	0.057081	-2.540577	0.000000
H	-2.228934	1.220395	-0.000000
H	-2.228934	-1.220395	-0.000000

C	1.311757	0.673789	0.000000
C	1.311757	-0.673789	0.000000
C	-0.072737	-1.472997	-0.000000
C	-0.072737	1.472997	-0.000000
C	-1.239030	-0.799318	-0.000000
C	-1.239030	0.799318	-0.000000
H	2.167594	-1.309287	0.000000
H	2.167594	1.309287	0.000000
H	0.049276	2.531001	0.000000
H	0.049276	-2.531001	0.000000
H	-2.216740	1.222388	-0.000000
H	-2.216740	-1.222388	-0.000000

C	1.295710	0.676411	0.000000
C	1.295710	-0.676411	0.000000
C	-0.062443	-1.460337	-0.000000
C	-0.062443	1.460337	-0.000000
C	-1.233283	-0.784051	-0.000000
C	-1.233283	0.784051	-0.000000
H	2.163039	-1.297721	0.000000
H	2.163039	1.297721	0.000000
H	0.041625	2.521253	0.000000
H	0.041625	-2.521253	0.000000
H	-2.204474	1.224159	-0.000000
H	-2.204474	-1.224159	-0.000000

C	1.279743	0.679084	0.000000
C	1.279743	-0.679084	0.000000
C	-0.052144	-1.447772	-0.000000
C	-0.052144	1.447772	-0.000000
C	-1.227621	-0.768827	-0.000000
C	-1.227621	0.768827	-0.000000
H	2.158258	-1.286215	0.000000
H	2.158258	1.286215	0.000000
H	0.034139	2.511340	0.000000
H	0.034139	-2.511340	0.000000
H	-2.192147	1.225704	-0.000000
H	-2.192147	-1.225704	-0.000000

C	1.263846	0.681811	0.000000
C	1.263846	-0.681811	0.000000
C	-0.041832	-1.435294	-0.000000
C	-0.041832	1.435294	-0.000000
C	-1.222041	-0.753636	-0.000000
C	-1.222041	0.753636	-0.000000
H	2.153254	-1.274781	0.000000
H	2.153254	1.274781	0.000000
H	0.026825	2.501272	0.000000
H	0.026825	-2.501272	0.000000
H	-2.179770	1.227021	-0.000000
H	-2.179770	-1.227021	-0.000000

C	1.248007	0.684597	0.000000
C	1.248007	-0.684597	0.000000
C	-0.031499	-1.422893	-0.000000
C	-0.031499	1.422893	-0.000000
C	-1.216539	-0.738466	-0.000000
C	-1.216539	0.738466	-0.000000
H	2.148031	-1.263428	0.000000
H	2.148031	1.263428	0.000000
H	0.019689	2.491056	0.000000
H	0.019689	-2.491056	0.000000
H	-2.167353	1.228108	-0.000000
H	-2.167353	-1.228108	-0.000000

C	1.232212	0.687444	0.000000
C	1.232212	-0.687444	0.000000
C	-0.021135	-1.410562	-0.000000
C	-0.021135	1.410562	-0.000000
C	-1.211113	-0.723303	-0.000000
C	-1.211113	0.723303	-0.000000
H	2.142592	-1.252166	0.000000
H	2.142592	1.252166	0.000000
H	0.012737	2.480700	0.000000
H	0.012737	-2.480700	0.000000
H	-2.154908	1.228964	-0.000000
H	-2.154908	-1.228964	-0.000000

C	1.216451	0.690356	0.000000
C	1.216451	-0.690356	0.000000
C	-0.010731	-1.398291	-0.000000
C	-0.010731	1.398291	-0.000000
C	-1.205761	-0.708137	-0.000000
C	-1.205761	0.708137	-0.000000
H	2.136942	-1.241005	0.000000
H	2.136942	1.241005	0.000000
H	0.005975	2.470214	0.000000
H	0.005975	-2.470214	0.000000
H	-2.142444	1.229588	-0.000000
H	-2.142444	-1.229588	-0.000000

C	1.200711	0.693338	0.000000
C	1.200711	-0.693338	0.000000
C	-0.000276	-1.386073	-0.000000
C	-0.000276	1.386073	-0.000000
C	-1.200479	-0.692953	-0.000000
C	-1.200479	0.692953	-0.000000
H	2.131089	-1.229954	0.000000
H	2.131089	1.229954	0.000000
H	-0.000591	2.459608	-0.000000
H	-0.000591	-2.459608	-0.000000
H	-2.129973	1.229981	-0.000000
H	-2.129973	-1.229981	-0.000000
